# False‐positive ^123^I‐metaiodobenzylguanidine scan in a patient with renal cell carcinoma: A case of chromophobe renal cell carcinoma oncocytic variant with a complicated clinical course

**DOI:** 10.1002/iju5.12233

**Published:** 2020-10-24

**Authors:** Takahito Suyama, Manato Kanesaka, Ayumi Fujimoto, Kotaro Otsuka, Kyokushin Hou, Kazuhiro Araki, Hiroshi Masuda, Satoko Kojima, Kazuto Yamazaki, Yukio Naya

**Affiliations:** ^1^ Department of Urology Teikyo University Chiba Medical Center Ichihara Japan; ^2^ Department of Pathology Teikyo University Chiba Medical Center Ichihara Chiba Japan; ^3^ Present address: Department of Urology Chiba University Graduate School of Medicine Chiba Japan

**Keywords:** ^123^I‐MIBG false‐positive, chromophobe RCC, oncocytic variant, Meigs’ syndrome

## Abstract

**Introduction:**

^123^I‐metaiodobenzylguanidine scanning has high sensitivity and specificity for the diagnosis of tumors derived from sympathetic nerves or the adrenal medulla. We report the rare case of a ^123^I‐metaiodobenzylguanidine false‐positive renal cell carcinoma.

**Case presentation:**

The patient was referred to our hospital with an incidental left renal mass during evaluation for hypertension. An ovarian tumor and prominent ascites were also observed. Serum and urine catecholamine levels were high to suspect a catecholamine‐producing tumor of the kidney. ^123^I‐metaiodobenzylguanidine scintigraphy showed increased ^123^I‐metaiodobenzylguanidine intake in the tumor. Laparoscopic radical left nephrectomy was performed. The pathologic diagnosis was an oncocytic variant of chromophobe renal cell carcinoma. No pheochromocytoma features were found.

**Conclusion:**

We report the first case of a ^123^I‐metaiodobenzylguanidine false‐positive renal cell carcinoma. This case was diagnosed with primary aldosteronism and Meigs’ syndrome, which made the clinical course more complicated.

**Abbreviations & Acronyms**
CT: computed tomography
HE: hematoxylin‐eosin
MIBG: metaiodobenzylguanidine
MRI: magnetic resonance imaging
PA: primary aldosteronism
RCC: renal cell carcinoma
SDH: succinate dehydrogenase

**Keynote message**

We report the first case of a ^123^I‐MIBG false‐positive RCC. ^123^I‐MIBG false positivity is a very rare phenomenon. We believe that our case will be helpful for clinicians who may encounter the same situation in the future.

## Case presentation

A 56‐year‐old woman was referred to our hospital with an incidental left renal mass found during evaluation for hypertension. Her height was 167 cm, and weight was 52 kg. Blood pressure was 166/102 mmHg, and heart rate was 116 bpm. She had a history of uncontrolled hypertension with amlodipine 10 mg once daily. She had a slight headache. On physical examination, there was significant ascites.

There were no abnormal results on general blood chemistry testing and blood counts. Serum catecholamine three fraction levels were high: adrenaline 148 (normal 0–100) pg/mL; noradrenaline 902 (normal 100–450) pg/mL; and dopamine 28 (normal 0–20) pg/mL. On 24‐h urine collection, adrenaline was 13.6 (normal 3.4–26.9) μg/day, noradrenaline was 250.6 (normal 48.6–168.4) μg/day, and dopamine was 1146 (normal 365–961.5) μg/day.

### Radiographic imaging

**Fig. 1:**
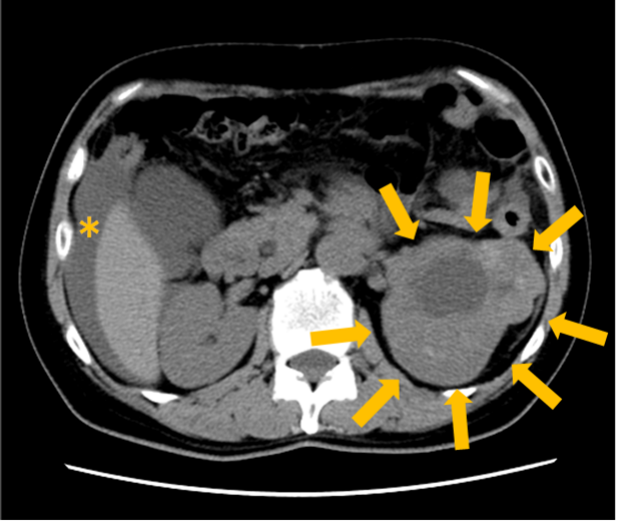
CT image. Arrow: left kidney tumor with internal degeneration. *Ascites.

CT showed a 11.5 × 8.6 cm tumor in the upper to middle pole of the left kidney, with a liquid component in the center (Fig. 1).

**Fig. 2:**
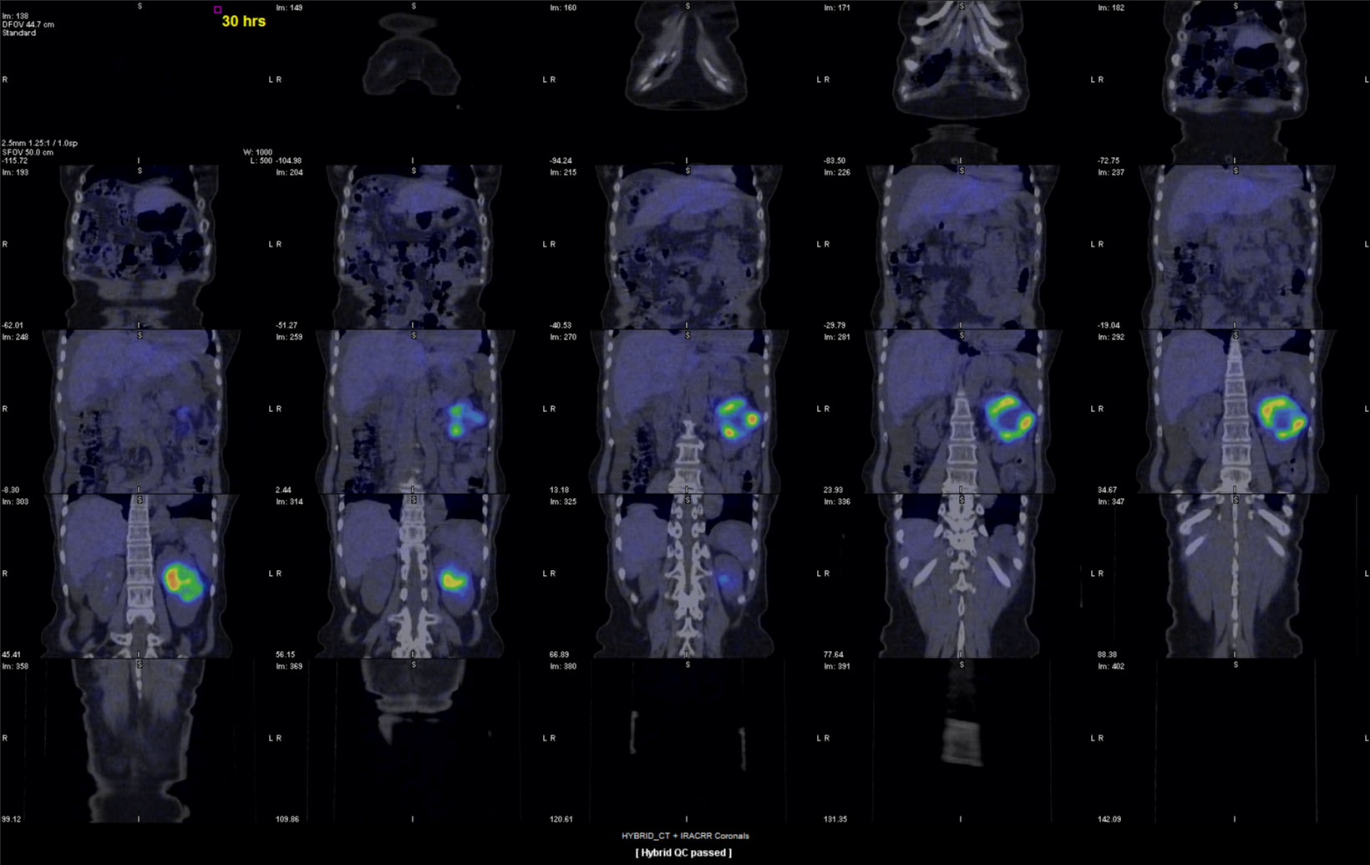
^123^I‐MIBG scintigraphy. ^123^I‐MIBG intake is increased in the left kidney tumor.

^123^I‐MIBG scintigraphy was performed and showed increased ^123^I‐MIBG intake in the tumor (Fig. 2).

On MRI, a capsule was detected on the kidney tumor with hypo‐ to iso‐intensity on T2‐weighted images. The diffusion activity was reduced in the tumor.

A 16.1 × 13.8 cm ovarian tumor was also observed. For the ovarian tumor, although a fibroma or fibrothecoma was first suspected based on the MRI images, metastasis of the left RCC could not be completely excluded.

The pre‐surgical diagnosis was a catecholamine‐producing tumor of the left kidney; considering the ascites and ovarian tumor, a high malignant potential tumor was suspected.

### Clinical course

With the diagnosis of a catecholamine‐producing left kidney tumor, an alpha‐blocker, doxazosin mesilate, was started at 4 mg/day. The dose of doxazosin mesilate was increased to 16 mg/day in 2 weeks. The hypertension was not controlled by the alpha‐blocker alone. Doxazosin mesilate 24 mg + amlodipine 10 mg + bisoprolol fumarate 2.5 mg were given to control hypertension.

During the clinical course, PA was diagnosed. Serum renin activity was 0.5 ng/mL/h, aldosterone concentration was 243 pg/mL, and the renin/aldosterone ratio was 486. A captopril test was positive. No nodule was detected in either adrenal gland. Naturally, localization was necessary, but an angiographic contrast agent could not be used due to the suspicion of a catecholamine‐producing tumor. Thus, we decided to perform surgery for the left kidney tumor first, before localization of the PA.

### Surgical findings

Laparoscopic radical left nephrectomy was performed. Operative time was 2 h 46 min. The removed left kidney weighed 420 g. Serous ascites of 3000 mL was present, and the ascites cytology was class II. Intraoperative blood pressure fluctuation due to manipulation of the tumor was not observed.

### Pathological findings and diagnosis

**Fig. 3:**
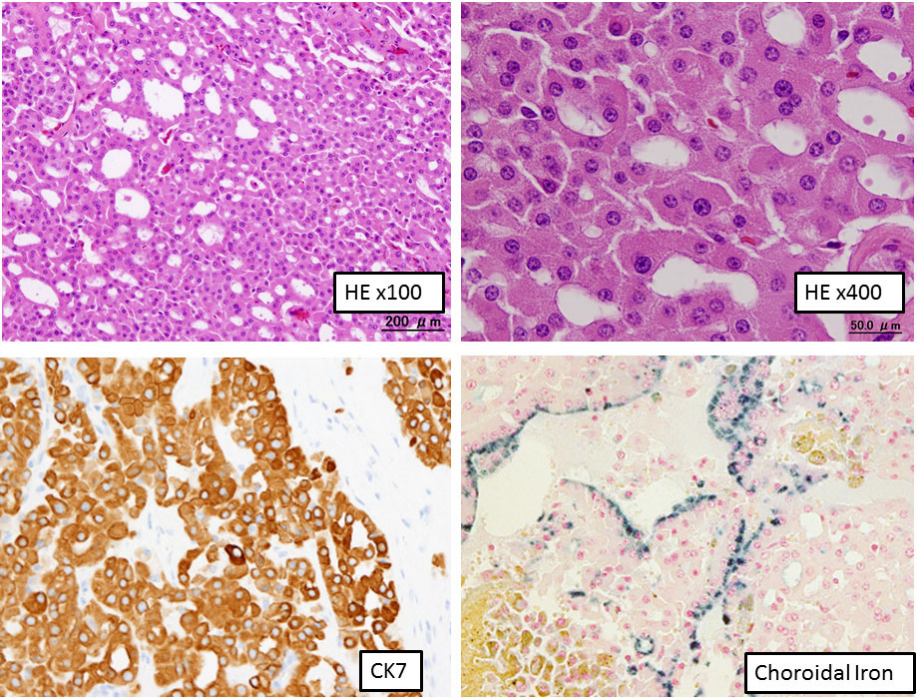
Microscopy, HE staining and immunohistochemistry. Eosinophilic cells with abundant granular cytoplasm are seen growing in a tubular and/or sheet*‐*like structure. The tumor was positive for CK7 and colloidal iron.

**Fig. 4:**
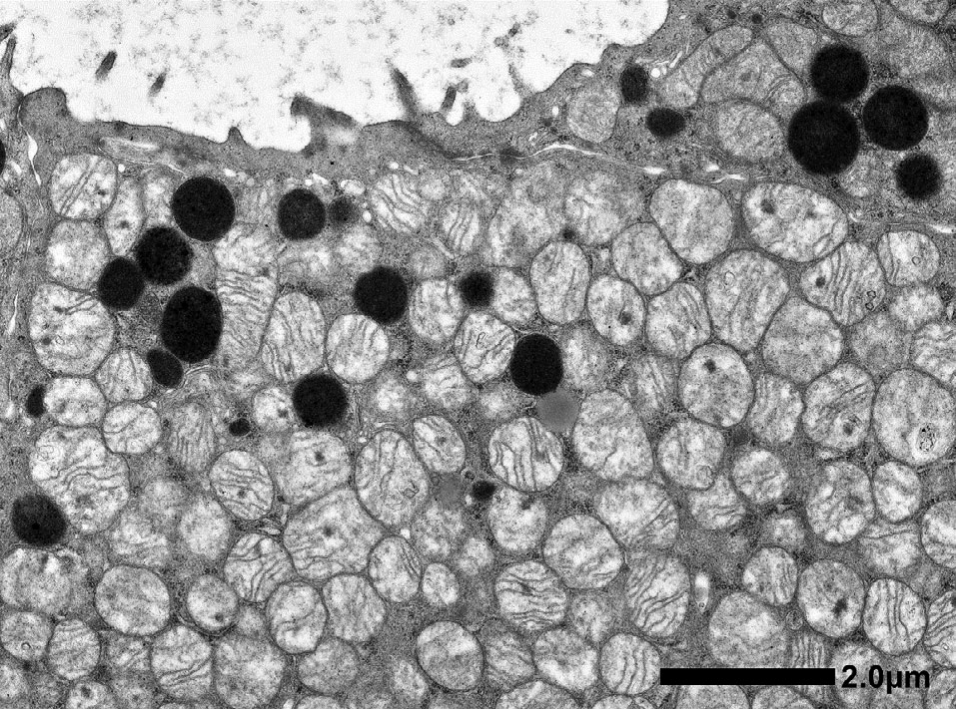
Electron microscopy. Abundant mitochondria are observed in the cytoplasm.

Microscopic findings showed that the tumor consisted of a tubular or sheet‐like growth pattern of cuboidal to low columnar cells with deeply eosinophilic cytoplasm (Fig. 3). Centrally located nuclei were uniformly round with prominent nucleoli. Arrangement of tumor cells in well‐defined solid nests separated by a loose hypocellular fibrous stroma, which is characteristic of renal oncocytoma was not observed. Involvement of perirenal fat was identified. The tumor showed positivity along the luminal sides for colloidal iron stain. Immunohistochemically, tumor cells showed diffuse positivity for CK7, c‐kit, and E‐cadherin, focally positive for vimentin, EMA, CD10, c‐kit, and RCC, but were negative for AMACR, Chromogranin A and synaptophysin (Fig. 3). Ultrastructure study showed numerous mitochondria with tubulocystic cristae in the cytoplasm of tumor cells (Fig. 4).

The tumor was diagnosed as a chromophobe RCC oncocytic variant.

### Postoperative course

Catecholamine levels decreased postoperatively to normal limits. Blood pressure was controlled with Nifedipine 20 mg and eplerenone 50 mg.

One month after surgery, an urgent right ovariectomy was performed due to ovarian torsion. On pathological examination, the diagnosis was a right ovarian fibroma. Meigs’ syndrome was diagnosed, and the ascites was found to be related to the ovarian fibroma.

## Discussion

^123^I‐MIBG scanning has high sensitivity and specificity for the diagnosis of pheochromocytoma.^1^ Considering the pre and post‐surgical serum catecholamine levels, it was suspected that the left RCC might be producing catecholamines. However, on pathological examination, there were no catecholamine‐producing pheochromocytoma cells. Tumor cells were also negative for neuroendocrine markers, and the presence of paraganglioma was ruled out. It was concluded that this was a ^123^I‐MIBG false‐positive RCC case.

Although false‐positive ^123^I‐MIBG accumulations are very rare, they have been reported in angiomyolipoma, hepatocellular carcinoma, accessory spleen, and adrenocortical carcinoma.^2^, ^3^, ^4^, ^5^ To the best of our knowledge, this is the first report of a ^123^I‐MIBG false‐positive RCC. There is a report that hydronephrosis was related to the accumulation of ^123^I‐MIBG in the kidney. In that case, ^123^I‐MIBG accumulation was observed in the renal cortex and urinoma in the kidney, in which hydronephrosis had been made by ureteric injury.^6^ In this case, the affected kidney showed hydronephrosis due to tumor, which may have resulted in false positivity of ^123^I‐MIBG.

This case was also diagnosed with Meigs’ syndrome. Meigs’ syndrome is a rare disorder of ovarian fibromas with ascites and pleural effusion.^7^ Ascites is attributed to transudation from the surface of the tumor due to direct pressure on surrounding lymphatics and vessels.^8^ The presence of ascites and ovarian tumor, coupled with increased uptake of ^123^I‐MIBG in kidney tumor, made us misidentify this tumor as highly malignant.

The pathological diagnosis of this case was a rare variant of RCC. It is characterized by morphologically oncocytoma‐like features and biologically chromophobe RCC‐like features.^9^, ^10^ In this case, the differential diagnosis with SDH‐deficient RCC is necessary. The mutations in SDH subunit are reported as a cause of familial paragangliomas/pheochromocytomas. SDH‐deficient RCC is characterized by eosinophilic cells with clear cytoplasmic inclusions and solid architecure.^11^ Morphologically, this case is different from the SDH‐deficient RCC. Immunostaining of CK7, which is negative in SDH‐deficient RCCs, was positive in this case. From these reasons, this case can be differentiated from an SDH‐deficient RCC.

The biology of this rare variant of RCC is not well understood. Further studies are warranted to clarify the characteristics of this tumor.

## Conflict of interest

The authors declare no conflict of interest.

## Author’s contribution

TS, MK: study concept and design. MK, AF, KO: acquisition of data. TS, KA: analysis and interpretation of data. TS: drafting of the manuscript. KH, HM, SK, KY, YN: critical revision of the manuscript for important intellectual content. YN: supervision.
